# Supplementary material for: Major depressive disorder and irritable bowel syndrome risk: A Mendelian randomization study
Source: PLoS One. 2024 Mar 14;19(3):e0300251. doi: 10.1371/journal.pone.0300251 (PMC10939280; doi:10.1371/journal.pone.0300251)
Supplement: S1 Table — (DOCX) [file pone.0300251.s001.docx]

**Table S1** Details of the GWASs included in the Mendelian randomization.

| Consortium | Phenotype | Participants | Web source |
| --- | --- | --- | --- |
| Psychiatric Genomics Consortium (PGC) | Major Depressive Disorder | 480,359 | <https://gwas.mrcieu.ac.uk/datasets/ieu-a-1187/> |
| Medical Research Council Integrative Epidemiology Unit (MRC-IEU) | irritable bowel syndrome | 462,933 | https://gwas.mrcieu.ac.uk/datasets/ukb-b-2592/ |
