# Supplementary material for: Major depressive disorder and irritable bowel syndrome risk: A Mendelian randomization study
Source: PLoS One. 2024 Mar 14;19(3):e0300251. doi: 10.1371/journal.pone.0300251 (PMC10939280; doi:10.1371/journal.pone.0300251)
Supplement: S2 Table — (DOCX) [file pone.0300251.s002.docx]

**Table S2** Single SNPs Used in the Two- sample Mendelian Randomization Analysis of the Causal

Effect of Major Depressive Disorder and Irritable Bowel Syndrome.

| No. | id.exposure | id.outcome | SNP | b | se | *p* |
| --- | --- | --- | --- | --- | --- | --- |
| 1 | ieu-a-1187 | ukb-b-2592 | rs10149470 | -0.004 | 0.0109 | 0.6999 |
| 2 | ieu-a-1187 | ukb-b-2592 | rs10950398 | 0.018 | 0.0116 | 0.1212 |
| 3 | ieu-a-1187 | ukb-b-2592 | rs10959913 | 0.0015 | 0.0108 | 0.889 |
| 4 | ieu-a-1187 | ukb-b-2592 | rs11135349 | 0.0185 | 0.0107 | 0.0846 |
| 5 | ieu-a-1187 | ukb-b-2592 | rs11643192 | 0.0106 | 0.012 | 0.3757 |
| 6 | ieu-a-1187 | ukb-b-2592 | rs11663393 | -0.006 | 0.0114 | 0.5694 |
| 7 | ieu-a-1187 | ukb-b-2592 | rs11682175 | -0.008 | 0.0113 | 0.467 |
| 8 | ieu-a-1187 | ukb-b-2592 | rs1226412 | 0.0028 | 0.0118 | 0.8132 |
| 9 | ieu-a-1187 | ukb-b-2592 | rs12552 | 0.0175 | 0.0074 | 0.0186 |
| 10 | ieu-a-1187 | ukb-b-2592 | rs12666117 | 0.02 | 0.0116 | 0.0839 |
| 11 | ieu-a-1187 | ukb-b-2592 | rs12958048 | 0.0137 | 0.0099 | 0.1645 |
| 12 | ieu-a-1187 | ukb-b-2592 | rs1354115 | -0.002 | 0.0118 | 0.892 |
| 13 | ieu-a-1187 | ukb-b-2592 | rs1432639 | 0.0192 | 0.0083 | 0.02 |
| 14 | ieu-a-1187 | ukb-b-2592 | rs159963 | 0.0185 | 0.0119 | 0.1184 |
| 15 | ieu-a-1187 | ukb-b-2592 | rs17727765 | 0.0109 | 0.0117 | 0.3498 |
| 16 | ieu-a-1187 | ukb-b-2592 | rs1806153 | 0.0123 | 0.0104 | 0.2353 |
| 17 | ieu-a-1187 | ukb-b-2592 | rs2005864 | 0.0122 | 0.0113 | 0.2808 |
| 18 | ieu-a-1187 | ukb-b-2592 | rs2389016 | 0.0015 | 0.0113 | 0.8977 |
| 19 | ieu-a-1187 | ukb-b-2592 | rs247910 | 0.0248 | 0.01 | 0.0136 |
| 20 | ieu-a-1187 | ukb-b-2592 | rs4074723 | 0.0056 | 0.0119 | 0.6378 |
| 21 | ieu-a-1187 | ukb-b-2592 | rs4904738 | 0.0165 | 0.0111 | 0.1345 |
| 22 | ieu-a-1187 | ukb-b-2592 | rs5758265 | 0.0259 | 0.0113 | 0.0211 |
| 23 | ieu-a-1187 | ukb-b-2592 | rs61867293 | -0.005 | 0.0107 | 0.6243 |
| 24 | ieu-a-1187 | ukb-b-2592 | rs6905391 | 0.0203 | 0.0096 | 0.0354 |
| 25 | ieu-a-1187 | ukb-b-2592 | rs7198928 | 0.0042 | 0.0115 | 0.7162 |
| 26 | ieu-a-1187 | ukb-b-2592 | rs7430565 | 0.011 | 0.0111 | 0.3197 |
| 27 | ieu-a-1187 | ukb-b-2592 | rs7856424 | -0.004 | 0.0114 | 0.6943 |
| 28 | ieu-a-1187 | ukb-b-2592 | rs8025231 | 0.017 | 0.0093 | 0.0687 |
| 29 | ieu-a-1187 | ukb-b-2592 | rs8063603 | 0.0033 | 0.011 | 0.7679 |
| 30 | ieu-a-1187 | ukb-b-2592 | rs915057 | 0.0034 | 0.0107 | 0.7473 |
| 31 | ieu-a-1187 | ukb-b-2592 | rs9427672 | 0.0103 | 0.0115 | 0.3702 |
| 32 | ieu-a-1187 | ukb-b-2592 | All - Inverse variance weighted | 0.0102 | 0.0019 | 1.01E-07 |
| 33 | ieu-a-1187 | ukb-b-2592 | All - MR Egger | 0.0281 | 0.0128 | 0.0368 |

Footnote: id.exposure represents the GWAS ID for MDD; id.outcome represents the GWAS ID for IBS; Samplesize: 462933
