# Supplementary material for: Major depressive disorder and irritable bowel syndrome risk: A Mendelian randomization study
Source: PLoS One. 2024 Mar 14;19(3):e0300251. doi: 10.1371/journal.pone.0300251 (PMC10939280; doi:10.1371/journal.pone.0300251)
Supplement: S3 Table — (DOCX) [file pone.0300251.s003.docx]

**Table S3** Leave-one-out sensitivity analysis of causal effects of Major Depressive Disorder and Irritable Bowel Syndrome.

| No. | id.exposure | id.outcome | SNP | b | se | *p* |
| --- | --- | --- | --- | --- | --- | --- |
| 1 | ieu-a-1187 | ukb-b-2592 | rs10149470 | 0.010677 | 0.001949 | 4.31E-08 |
| 2 | ieu-a-1187 | ukb-b-2592 | rs10950398 | 0.009997 | 0.001945 | 2.77E-07 |
| 3 | ieu-a-1187 | ukb-b-2592 | rs10959913 | 0.010499 | 0.00195 | 7.25E-08 |
| 4 | ieu-a-1187 | ukb-b-2592 | rs11135349 | 0.009941 | 0.00195 | 3.43E-07 |
| 5 | ieu-a-1187 | ukb-b-2592 | rs11643192 | 0.010205 | 0.001944 | 1.52E-07 |
| 6 | ieu-a-1187 | ukb-b-2592 | rs11663393 | 0.010703 | 0.001946 | 3.83E-08 |
| 7 | ieu-a-1187 | ukb-b-2592 | rs11682175 | 0.010765 | 0.001947 | 3.23E-08 |
| 8 | ieu-a-1187 | ukb-b-2592 | rs1226412 | 0.010418 | 0.001945 | 8.45E-08 |
| 9 | ieu-a-1187 | ukb-b-2592 | rs12552 | 0.009695 | 0.001986 | 1.05E-06 |
| 10 | ieu-a-1187 | ukb-b-2592 | rs12666117 | 0.009938 | 0.001945 | 3.25E-07 |
| 11 | ieu-a-1187 | ukb-b-2592 | rs12958048 | 0.010077 | 0.001956 | 2.57E-07 |
| 12 | ieu-a-1187 | ukb-b-2592 | rs1354115 | 0.010533 | 0.001944 | 6.04E-08 |
| 13 | ieu-a-1187 | ukb-b-2592 | rs1432639 | 0.009702 | 0.001973 | 8.74E-07 |
| 14 | ieu-a-1187 | ukb-b-2592 | rs159963 | 0.009992 | 0.001944 | 2.76E-07 |
| 15 | ieu-a-1187 | ukb-b-2592 | rs17727765 | 0.010196 | 0.001945 | 1.59E-07 |
| 16 | ieu-a-1187 | ukb-b-2592 | rs1806153 | 0.01014 | 0.001952 | 2.06E-07 |
| 17 | ieu-a-1187 | ukb-b-2592 | rs2005864 | 0.010157 | 0.001947 | 1.82E-07 |
| 18 | ieu-a-1187 | ukb-b-2592 | rs2389016 | 0.010474 | 0.001947 | 7.45E-08 |
| 19 | ieu-a-1187 | ukb-b-2592 | rs247910 | 0.009663 | 0.001955 | 7.67E-07 |
| 20 | ieu-a-1187 | ukb-b-2592 | rs4074723 | 0.010339 | 0.001944 | 1.05E-07 |
| 21 | ieu-a-1187 | ukb-b-2592 | rs4904738 | 0.010018 | 0.001948 | 2.71E-07 |
| 22 | ieu-a-1187 | ukb-b-2592 | rs5758265 | 0.009744 | 0.001947 | 5.61E-07 |
| 23 | ieu-a-1187 | ukb-b-2592 | rs61867293 | 0.010726 | 0.00195 | 3.79E-08 |
| 24 | ieu-a-1187 | ukb-b-2592 | rs6905391 | 0.0098 | 0.001958 | 5.57E-07 |
| 25 | ieu-a-1187 | ukb-b-2592 | rs7198928 | 0.010388 | 0.001946 | 9.39E-08 |
| 26 | ieu-a-1187 | ukb-b-2592 | rs7430565 | 0.010189 | 0.001948 | 1.69E-07 |
| 27 | ieu-a-1187 | ukb-b-2592 | rs7856424 | 0.010642 | 0.001946 | 4.56E-08 |
| 28 | ieu-a-1187 | ukb-b-2592 | rs8025231 | 0.009916 | 0.00196 | 4.23E-07 |
| 29 | ieu-a-1187 | ukb-b-2592 | rs8063603 | 0.010432 | 0.001948 | 8.59E-08 |
| 30 | ieu-a-1187 | ukb-b-2592 | rs915057 | 0.010441 | 0.00195 | 8.63E-08 |
| 31 | ieu-a-1187 | ukb-b-2592 | rs9427672 | 0.010212 | 0.001946 | 1.54E-07 |
| 32 | ieu-a-1187 | ukb-b-2592 | All | 0.010215 | 0.001919 | 1.01E-07 |

Footnote: id.exposure represents the GWAS ID for MDD; id.outcome represents the GWAS ID for IBS; Samplesize: 462933
