# Supplementary material for: Major depressive disorder and irritable bowel syndrome risk: A Mendelian randomization study
Source: PLoS One. 2024 Mar 14;19(3):e0300251. doi: 10.1371/journal.pone.0300251 (PMC10939280; doi:10.1371/journal.pone.0300251)
Supplement: S1 Fig — (DOCX) [file pone.0300251.s004.docx]

**Figure S1** Funnel plot of single SNPs used in the Mendelian randomization analysis of the effects of Major Depressive Disorder and Irritable Bowel Syndrome.

**
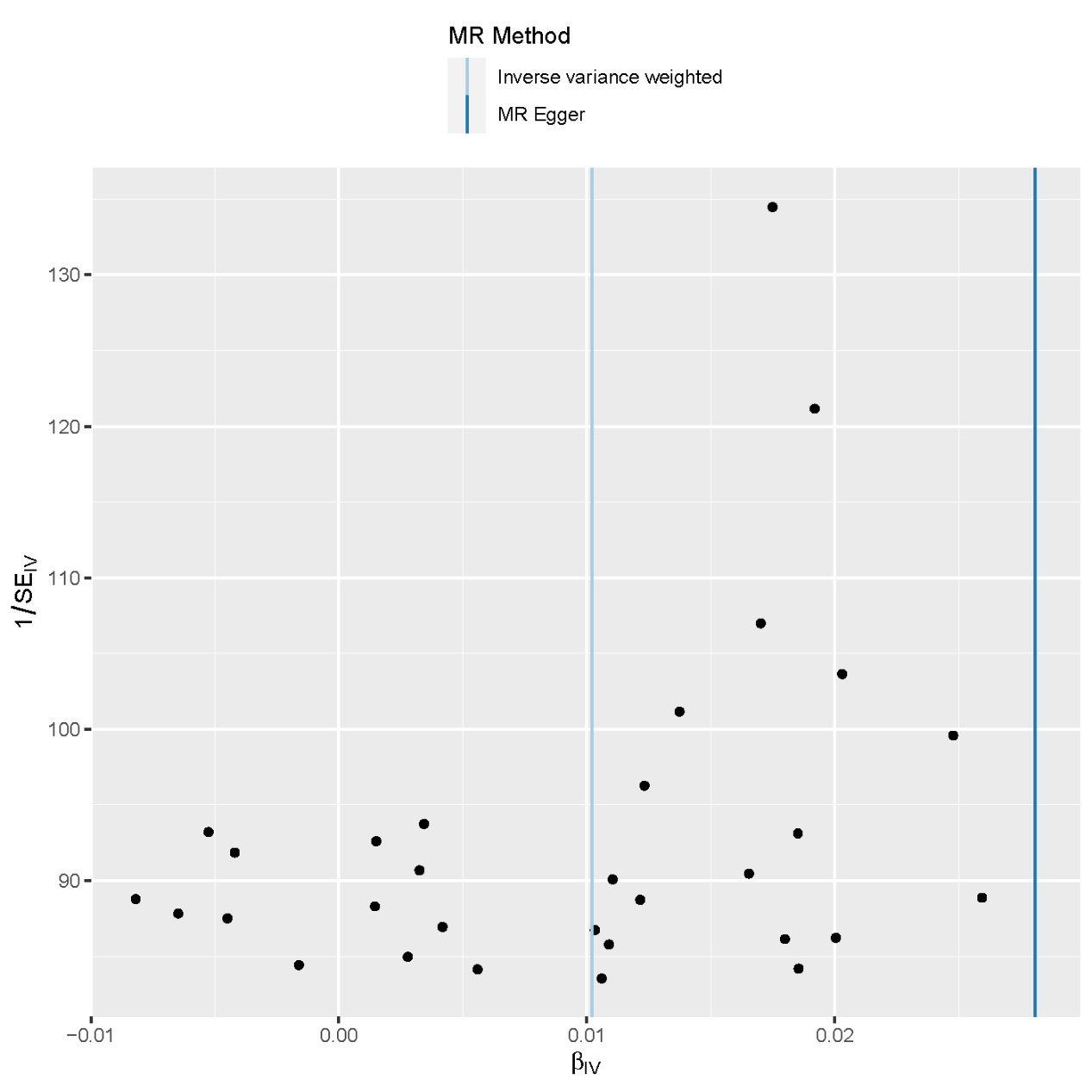
**
