# Supplementary material for: Major depressive disorder and irritable bowel syndrome risk: A Mendelian randomization study
Source: PLoS One. 2024 Mar 14;19(3):e0300251. doi: 10.1371/journal.pone.0300251 (PMC10939280; doi:10.1371/journal.pone.0300251)
Supplement: Attachment — Submitted filename: PLOS_ONE_Article_review.docx [file pone.0300251.s006.docx]

**Title:** Major depressive disorder and irritable bowel syndrome risk: A Mendelian randomization study

This paper utilizes Mendelian randomization to explore the potential causal relationship between major depressive disorder (MDD) and irritable bowel syndrome (IBS). Leveraging data from the Psychiatric Genomics Consortium and the Medical Research Council Integrative Epidemiology Unit, the study employs various Mendelian randomization methods, revealing a significant genetic link between MDD and an elevated risk of IBS. Rigorous analyses, including assessments for heterogeneity and bidirectionality, support the study's findings. Notably, the absence of a genetic link between inflammatory bowel disease and MDD is observed. In conclusion, the study underscores a direct causal association between MDD and IBS, offering valuable insights for clinical management. By incorporating the following change, the paper can potentially be further improved.

**Abstract**

1. In the Result section of Abstract author using first time IVW in the paper consider defining its full form here
    IVW method 🡪	Inverse Variance Weighting (IVW) method

**Introduction**

1. "but there is no known structural abnormalities" might read better as "but there are no known structural abnormalities."
2. "According to a systematic study released in2013" should have a space between "in" and "2013" for proper formatting.
3. "This patients had greater rates of IBS symptoms than those in the control group" needs to be corrected to "These patients had higher rates of IBS symptoms than those in the control group."
4. “ According to a systematic study released in2013, the cost of irritable bowel syndrome treatment ranges from $1,562 to $7,547 year for direct costs and from $791 to $7,737 annually for indirect costs in the United States[10].” This sentence is not clear. Is treatment ranges from $1,562 to $7,547 per year?

**Methods**

The methods section provides a comprehensive approach to Mendelian randomization (MR) but could benefit from a clearer organization and improved clarity in several areas:

1. The three main hypotheses for an MR study could be presented more clearly, perhaps with a brief explanation for each, ensuring that the reader grasps their significance to the study.
2. The section on data resources is concise but consider providing a brief rationale for choosing the Psychiatric Genomics Consortium (PGC) and Medical Research Council Integrative Epidemiology Unit (MRC-IEU) datasets, highlighting their relevance to the research.
3. The criteria for SNP selection are well-defined, but it might be beneficial to provide a concise justification for these criteria and their significance in the context of the study.
4. The mention of the TwoSampleMR and MRPRESSO packages lacks supportive information or citation.
5. The phrase "version0.5.6" should have a space between "version" and the version number.

**Results**

1. The author states that in this study, genome-wide significant and independent SNPs were chosen as instrumental variables (IVs) for Mendelian Randomization (MR) analysis. However, the text lacks a clear explanation of how these criteria enhance the reliability of the instrumental variables. Providing a concise clarification on how the stringent criteria for significance and independence contribute to the robustness and validity of the MR analysis would strengthen the clarity and transparency of the methodology.
2. **Sensitivity Analysis:** Elaborate briefly on why these specific sensitivity analyses were chosen and how they contribute to the robustness of the main analysis.
3. **Leave-One-Out Sensitivity Analysis:** Consider incorporating a brief interpretation of the results of this analysis.
4. The inclusion of a leave-one-out sensitivity analysis is commendable, providing insight into the stability of the causal effect estimation. However, to enhance clarity, it would be beneficial to briefly define or explain what a forest map is for readers unfamiliar with the term. Additionally, consider specifying the key findings or patterns observed in the forest map that support the conclusion of a reliable and stable causal effect of MDD on IBS, even when individual SNPs are excluded. This would provide a more comprehensive and accessible interpretation of the results for a broader audience.
5. **Quality control:** Providing a concise explanation of each method (Horizontal polymorphism, heterogeneity analyses, and sensitivity analyses ) and their relevance to quality control would enhance transparency and the overall robustness of the study.

**Discussion**

The discussion provides valuable insights into the association between Major Depressive Disorder (MDD) and Irritable Bowel Syndrome (IBS) using Mendelian randomization (MR) analysis and large-scale GWAS data. It highlights the significance of the gut-brain axis in connecting gut and brain processes and suggests a causal link between MDD and IBS.

1. However, the discussion could be strengthened by incorporating references to support the mentioned hypotheses and findings. It's essential to provide citations for past studies or theories that have contributed to the understanding of the gut-brain axis and the relationship between MDD and IBS. Referencing existing literature would add credibility to the claims made in the discussion.
2. Finally, the limitations are appropriately acknowledged, but it would be beneficial to elaborate on potential biases or confounding factors that might affect the generalizability of the results. This would add nuance to the interpretation of the study's limitations.
3. The phrase "It is important to prevent IBS among MDD patients at the oneset" contains a typographical error. It should be "onset" instead of "oneset."

**Conclusion**

1. The discussion effectively emphasizes the clinical implications of understanding the causal relationship between MDD and IBS, advocating for prevention and control strategies. However, explicitly stating the potential clinical impact of the study's findings on patient care and management would provide a more concrete conclusion.
