# Supplementary material for: Major depressive disorder and irritable bowel syndrome risk: A Mendelian randomization study
Source: PLoS One. 2024 Mar 14;19(3):e0300251. doi: 10.1371/journal.pone.0300251 (PMC10939280; doi:10.1371/journal.pone.0300251)
Supplement: Attachment — Submitted filename: Response to Reviewers.docx [file pone.0300251.s007.docx]

Thank the Editor and reviewers for the time and effort that you have put into reviewing the revised version of the manuscript. Your suggestions have enabled us to improve our work. Based on the instructions provided in your letter, we made the corresponding modification carefully.

| **suggestion** | **response to the suggestion** | **where the change was made in the manuscript, table, or figures** |
| --- | --- | --- |
| **Editor** |  |  |
| Please describe the validity of the key assumptions for the MR analysis, especially the effect of potential confounders. | Thank you for your suggestion. Following your advice, I have rephrased the validity of the key assumptions for the MR analysis and elaborated on the impact of potential confounders in the Methods section. Modification as follows:  Our study utilized the TSMR method to examine the relationship between MDD and IBS. We based our MR analysis on three fundamental hypotheses: (1) Correlation hypothesis: The chosen genetic variant should demonstrate a strong association with the exposure variable. Weak instruments may result in biased estimates; (2) Independence hypothesis: This hypothesis suggests that the genetic variant used as an instrument for the exposure variable is associated with the outcome solely through its impact on the exposure, without any alternative pathway involvement. In essence, the genetic variant should not be linked to any confounders of the exposure-outcome relationship; (3) Exclusion hypothesis: This hypothesis asserts that the genetic variant used as an instrument influences the outcome exclusively through its effect on the exposure, without involvement in any other pathway. Violation of this assumption, such as pleiotropy (wherein the genetic variant affects multiple phenotypes), can lead to biased estimates. In traditional observational studies, confounders are variables associated with both the exposure and outcome of interest. Failure to properly control for confounders can result in biased estimates of the exposure-outcome relationship. Mendelian randomization mitigates confounding factors by employing genetic variants as instrumental variables (IVs). These IVs are randomly allocated during meiosis and are less susceptible to environmental or lifestyle influences. This approach mimics a randomized controlled trial, leveraging the natural randomization of genetic inheritance to reduce bias from confounding variables. | Lines 79-97 |
| While the results are statistically significant, the effect size found is rather small (i.e., OR=1.01). | Thank you for your kind advice. I have carefully revised the manuscript according to your suggestions. Explanation as follows:  “Mendelian randomization is primarily used to assess causal relationships rather than directly determine the magnitude of quantitative effect sizes. While Mendelian randomization can provide insights into the direction and plausibility of the causal impact of an exposure on an outcome, it often cannot precisely measure the magnitude of this effect. Mendelian randomization utilizes genetic variants to randomly allocate exposure, mimicking the design of a randomized controlled trial. Because genetic variants are randomly assigned during the process of inheritance, their relationship with the exposure is less likely to be influenced by confounding factors. Therefore, Mendelian randomization studies can provide more reliable inference about the causal impact of exposure on the outcome. Although Mendelian randomization can offer clues about causal relationships, its results typically do not directly yield quantitative effect sizes. Assessing the magnitude of effect sizes may require incorporating other types of study designs, such as cohort studies or randomized controlled trials, to obtain more precise estimates. Thus, when interpreting Mendelian randomization study results, the emphasis is usually placed on evaluating the presence and direction of causal relationships rather than directly quantifying the size of effects.” | Lines 172-186. |
| What are the columns of id.exposure and id.outcome in Tables S2 and S3? Please annotate them appropriately. Also, the column of sample size is not needed, which can be briefly described as a footnote. | Thank you for your kind advice.  One phenotype corresponds to multiple datasets, where id.exposure and id.outcome represent the GWAS IDs utilized in our Mendelian analysis. In particular, id.exposure represents the GWAS ID for MDD and id.outcome represents the GWAS ID for IBS.  Following your advice, I have deleted the column of sample size provided a brief explanation for the above content in the footnote. |  |
| Minor comments: (1) the MAF threshold for SNP selection should be “>=0.3” but not “=0.3”; (2) please clarify whether R2 of 0.1 or 0.001 was used for LD threshold; (3) please explain the dots in Figure 2. | Thank you for your kind advice.  (1) the MAF threshold for SNP selection has been modified to be “>=0.3”  (2) R2 of 0.001 was used for LD threshold.  And I have made the corresponding modification in the Methods and Results sections of the article, as follows:  “To assess LD between the included SNPs, a clumping process was conducted (R²<0.001 and clumping distance = 10,000 kb).”  “LD between SNPs was evaluated using data from the 1000 Genomes Project European samples, and only SNPs with an LD value of R²<0.001 (clumping window size=10,000kb) and the lowest P-values were retained.”  (3) Each data point corresponds to a SNP serving as an instrumental variable, with the accompanying line indicating the 95% confidence interval.  And I have made the corresponding modification in the Methods section of the article, as follows:  “Method comparison plot (Fig.2) represents the results of Mendelian randomization (MR) analysis between MDD and IBS. Each data point corresponds to a SNP serving as an instrumental variable, with the accompanying line indicating the 95% confidence interval. The x-axis denotes the SNP's effect on the exposure factor (MDD), while the y-axis indicates its effect on the outcome factor (IBS). The slope of the colored lines reflects the ratio of these effects, representing the exposure's impact on the outcome. Different colors represent distinct algorithms. Overall, the lines exhibit an upward trend, suggesting that as MDD increases, the risk of IBS also rises.” | (1) Line 166. (2) Lines 128-129; lines 163-165. (3) Lines 193-200. |
| **Reviewer1** |  |  |
| The authors have explored the causal association of major depressive disorder with irritable bowel syndrome through a bidirectional mendelian randomization study. The design of this study is reasonable, and the manuscript language needs to be further adjusted before publication. | Thank you for your suggestion. I have already made further adjustments to the language of the manuscript. |  |
| **Reviewer2** |  |  |
| (Exclusion hypothesis) (Figs.1). Figs 1 to be written as Fig 1. | Thank you for your kind advice. I have carefully revised the manuscript according to your suggestions. | Line 80. |
| Table 1, P val or P or italicized p to be to be standardized throughout the manuscript. 95% to be written as 95% CI. | Thank you for your kind advice. I have carefully revised the manuscript according to your suggestions. | Line 24.  Line 138.  Line 162.  Line 227.  Line 238.  Table 1. |
| ( Supplementary Tables S1 to be presented as (Supplementary Table S1). For year[1], the gap of the cited reference to be spaced out. This apply to others. | Thank you for your kind advice. I have carefully revised the manuscript according to your suggestions. | Line 115.  Article. |
| Labelled to be written in the first column for Table S2 and Table S3. | Thank you for your kind advice. I have carefully revised the manuscript according to your suggestions. | Table S2 and Table S3. |
| The sentence ‘… individual SNP IVs: MR-Egger regression….’ requires revision. | Thank you for your kind advice. I have made the corresponding modification in the Methods section of the article, as follows:  “In addition, a sensitivity analysis was conducted to assess potential bias resulting from directional pleiotropy or heterogeneity of individual SNP IVs. This involved employing MR-Egger regression, weighted median, and weighted mode MR methods as supplements to the IVW method.” | Line 139-142. |
| The definition of IVW could be improved e.g. The inverse variance weighted (IVW) method estimates a causal effect by calculating the slope of a regression line over the associations between the weighted SNP-mean exposure and SNP-mean outcome (oriented to be positive). | Thank you for your kind advice. I have carefully revised the manuscript according to your suggestions. | Line 135-137. |
| For Figure 2, the figure in X and Y axis to be labelled. | Thank you for your feedback. Following your advice, I have labeled the X-axis and Y-axis on Figure 2. | Figure 2. |
| p < 5.0 x 10-8 incorrectly labelled. | Thank you for your kind advice. I have carefully revised the manuscript according to your suggestions. | Line 162. |
| The format presentation for (p < 5.0 x 10^-8) to follow the format that were presented in Supplementary Tables e.g. p <5.0E-08. | Thank you for your kind advice. I have carefully revised the manuscript according to your suggestions. | Line 124. |
| References did not conform to the journal format. | Thank you for your kind advice. I have carefully revised the manuscript according to your suggestions. | References. |
| **Reviewer3** |  |  |
| 1. In the Result section of Abstract author using first time IVW in the paper consider defining its full form here  IVW method Inverse Variance Weighting (IVW) method | Thank you for your kind advice. I have carefully revised the manuscript according to your suggestions. Modification as follows:  “In the standard Inverse Variance Weighting (IVW) method”. | Line 20. |
| Introduction  1. "but there is no known structural abnormalities" might read better as "but there are no known structural abnormalities." | Thank you for your kind advice. I have carefully revised the manuscript according to your suggestions. Modification as follows:  "but there are no known structural abnormalities." | Line 44. |
| Introduction  2. "According to a systematic study released in2013" should have a space between "in" and "2013" for proper formatting. | Thank you for your kind advice. I have carefully revised the manuscript according to your suggestions. Modification as follows:  “According to a systematic study released in 2013” | Lines 50-51. |
| Introduction  3. "This patients had greater rates of IBS symptoms than those in the control group" needs to be corrected to "These patients had higher rates of IBS symptoms than those in the control group. | Thank you for your kind advice. I have carefully revised the manuscript according to your suggestions. Modification as follows:  "These patients had higher rates of IBS symptoms than those in the control group.” | Lines 58-59. |
| Introduction  4. “According to a systematic study released in2013, the cost of irritable bowel syndrome treatment ranges from $1,562 to $7,547 year for direct costs and from $791 to $7,737 annually for indirect costs in the United States [10].” This sentence is not clear. Is treatment ranges from $1,562 to $7,547 per year? | Thank you for your kind advice. I have carefully revised the manuscript according to your suggestions. Modification as follows:  “According to a systematic study released in 2013, the cost of irritable bowel syndrome treatment ranges from $1,562 to $7,547 per year for direct costs and from $791 to $7,737 annually for indirect costs in the United States.” | Lines 51-52. |
| Methods 1. The three main hypotheses for an MR study could be presented more clearly, perhaps with a brief explanation for each, ensuring that the reader grasps their significance to the study. | Thank you for your kind advice. I have carefully revised the manuscript according to your suggestions. Modification as follows: | Lines 78-89. |
| Methods  2. The section on data resources is concise but consider providing a brief rationale for choosing the Psychiatric Genomics Consortium (PGC) and Medical Research Council Integrative Epidemiology Unit (MRC-IEU) datasets, highlighting their relevance to the research. | Thank you for your kind advice. I have carefully revised the manuscript according to your suggestions. Modification as follows:  “The Psychiatric Genomics Consortium (PGC) is recognized as a pioneering initiative in psychiatry, fostering collaboration and accelerating progress in unraveling the genetic basis of psychiatric disorders. With over 800 investigators spanning 36 countries and a participant cohort exceeding 400,000 individuals, the PGC represents a global effort to advance mental health research.”  “The Medical Research Council (MRC) Integrative Epidemiology Unit at the University of Bristol (MRC IEU) aims to integrate molecular, cellular, clinical, and population data to identify causal associations between modifiable exposures and health outcomes. Employing a multidisciplinary approach, the Unit addresses key public health issues pertinent to both the UK and global agendas. By leveraging disciplines beyond traditional epidemiology, the MRC IEU enhances causal and translational epidemiology.” | Lines 99-103.  Lines 105-111. |
| Methods  3. The criteria for SNP selection are well-defined, but it might be beneficial to provide a concise justification for these criteria and their significance in the context of the study. | Thank you for your kind advice. I have carefully revised the manuscript according to your suggestions. Modification as follows:  “To ensure the authenticity and accuracy of the conclusions regarding the causal relationship between MDD and IBS, rigorous quality control measures were implemented to select optimal instrumental variables (IVs). Firstly, SNPs significantly associated with MDD were chosen as IVs. Two thresholds were applied for IV selection. Initially, SNPs with p-values below the genome-wide statistical significance threshold (5.00E-08) were selected to serve as IVs. Secondly, a minor allele frequency (MAF) threshold of 0.3 was applied to the variants of interest. Thirdly, adherence to one of the principles of the MR approach, which dictates that there should be no linkage disequilibrium (LD) among the included IVs, was ensured. To assess LD between the included SNPs, a clumping process was conducted (R²<0.001 and clumping distance = 10,000 kb). Fourthly, a crucial step in MR analysis is to confirm that the effects of the SNPs on the exposure align with the same allele as the effects on the outcome. To prevent distortion of strand orientation or allele coding, palindromic SNPs (e.g., those with A/T or G/C alleles) were excluded.” | Lines 118-130. |
| Methods  4. The mention of the TwoSampleMR and MRPRESSO packages lacks supportive information or citation. | Thank you for your kind advice. I have carefully revised the manuscript according to your suggestions. Modification as follows:  “Analyses were implemented by the package TwoSampleMR (version 0.5.6) [18] and MRPRESSO (version 1.0) [19] in R (version 4.0.1). No additional ethical approval was required due to the re-analysis of previously collected and published data.” | Lines 152-153. |
| Methods  5. The phrase "version0.5.6" should have a space between "version" and the version number. | Thank you for your kind advice. I have carefully revised the manuscript according to your suggestions. Modification as follows:  “version 0.5.6”. | Line 153. |
| Results  1. The author states that in this study, genome-wide significant and independent SNPs were chosen as instrumental variables (IVs) for Mendelian Randomization (MR) analysis. However, the text lacks a clear explanation of how these criteria enhance the reliability of the instrumental variables. Providing a concise clarification on how the stringent criteria for significance and independence contribute to the robustness and validity of the MR analysis would strengthen the clarity and transparency of the methodology. | Thank you for your kind advice. I have carefully revised the manuscript according to your suggestions. Modification as follows:  “In this study, instrumental variables (IVs) were selected using specific criteria. Single nucleotide polymorphisms (SNPs) associated with each trait at the locus-wide significance threshold (p < 5.00E-08) were considered as potential IVs to ensure a robust correlation between genetic variants and the exposure variable. LD between SNPs was evaluated using data from the 1000 Genomes Project European samples, and only SNPs with an LD value of R²<0.001 (clumping window size=10,000kb) and the lowest P-values were retained. Additionally, SNPs with a MAF ＞=0.3 were excluded to ensure the independence of the genetic variables. Ultimately, 31 statistically significant SNPs were identified from GWAS summary data for MR analysis (Supplementary Table S2).” | Lines 158-166. |
| Results  2. Sensitivity Analysis: Elaborate briefly on why these specific sensitivity analyses were chosen and how they contribute to the robustness of the main analysis. | Thank you for your kind advice. I have carefully revised the manuscript according to your suggestions. Modification as follows:  “We also performed MR-Egger, Weighted median, and Weighted mode for sensitivity analysis to verify the consistency of test results (Table 1). The MR-Egger method tested the null association hypothesis and provided an effect estimate, considering potential invalid SNPs [17]. The weighted median approach, providing a consistent estimate even with invalid SNPs [15]. The weighted mode method can offer a robust causal estimate when the majority of individual estimates were from valid IVs [15].” | Lines 188-193. |
| Results  3. Leave-One-Out Sensitivity Analysis: Consider incorporating a brief interpretation of the results of this analysis. | Thank you for your kind advice. I have carefully revised the manuscript according to your suggestions. Modification as follows:  “The forest plot revealed a reliable and stable outcome, indicating that the causal effect of MDD on IBS remained consistent and was not significantly affected by excluding any single SNP.” | Lines 205-207. |
| Results  4. The inclusion of a leave-one-out sensitivity analysis is commendable, providing insight into the stability of the causal effect estimation. However, to enhance clarity, it would be beneficial to briefly define or explain what a forest map is for readers unfamiliar with the term. Additionally, consider specifying the key findings or patterns observed in the forest map that support the conclusion of a reliable and stable causal effect of MDD on IBS, even when individual SNPs are excluded. This would provide a more comprehensive and accessible interpretation of the results for a broader audience. | Thank you for your kind advice. I have carefully revised the manuscript according to your suggestions. Modification as follows:  “In the final step, we conducted leave-one-out sensitivity analysis to determine if the association between MDD and IBS was disproportionately influenced by any single SNP. The resulting forest plot was then examined (Supplementary Figure S2). Each black point in the forest plot represents the MR analysis (using IVW) excluding that particular SNP. Furthermore, the overall analysis including all SNPs is presented for comparison. The forest plot revealed a reliable and stable outcome, indicating that the causal effect of MDD on IBS remained consistent and was not significantly affected by excluding any single SNP.” | Lines 200-207. |
| Results  5. Quality control: Providing a concise explanation of each method (Horizontal polymorphism, heterogeneity analyses, and sensitivity analyses) and their relevance to quality control would enhance transparency and the overall robustness of the study. | Thank you for your kind advice. I have carefully revised the manuscript according to your suggestions. Modification as follows:  “In order to conduct quality control, in addition to performing the leave-one out sensitivity, we also conducted horizontal polymorphism and heterogeneity analysis. The MR-Egger intercept test evaluated pleiotropy, with a significant difference from zero indicating potential horizontal pleiotropy. If SNPs influence the outcome through a pathway other than the exposure, it constitutes horizontal pleiotropy, violating Mendelian randomization (MR) assumptions. Consistent bias in MR estimates can result if the average horizontal pleiotropic effect of SNPs favors one direction. The Egger regression intercept provides an estimate of horizontal pleiotropy magnitude.”  “Heterogeneity pertains to the variability in causal estimates obtained for each SNP, indicating the consistency of the causal estimate across all SNPs. Lower heterogeneity implies greater reliability of Mendelian randomization (MR) estimates. Heterogeneity was evaluated using each applicable MR method. Heterogeneity between SNPs was assessed using Cochran's Q statistics.” | Lines 209-216.  Lines 224-229. |
| Discussion  1. However, the discussion could be strengthened by incorporating references to support the mentioned hypotheses and findings. It's essential to provide citations for past studies or theories that have contributed to the understanding of the gut-brain axis and the relationship between MDD and IBS. Referencing existing literature would add credibility to the claims made in the discussion. | Thank you for your kind advice. I have carefully revised the manuscript according to your suggestions. Modification as follows:  “A potential mechanism that could explain the increased risk of IBS associated with MDD involves the activation of the cingulate region of the limbic system in the cerebral cortex. Drossman et al. utilized functional magnetic resonance imaging and observed that depression can elicit activity in this brain region [25]; Importantly, the limbic system's emotional activity center shares the same anatomical site as the vegetative and endocrine regulatory centers, which govern digestive tract movement and secretion. Consequently, alterations in the function of these centers may occur, leading to changes in neurotransmitters and adrenocorticotropin-releasing factors. These alterations can subsequently impact visceral sensation, intestinal movement, and endocrine function. Thus, this cascade of events may contribute to the onset or exacerbation of IBS symptoms [26].” | Lines 267-277. |
| Discussion  2. Finally, the limitations are appropriately acknowledged, but it would be beneficial to elaborate on potential biases or confounding factors that might affect the generalizability of the results. This would add nuance to the interpretation of the study's limitations. | Thank you for your kind advice. I have carefully revised the manuscript according to your suggestions. Modification as follows:  “There are several limitations to our study that should be noted. Firstly, in order to minimize bias related to population stratification, our study was restricted to individuals of European ancestry, limiting the generalizability of our findings to other ancestral backgrounds. Secondly, the identification of IBS cases relied on self-reported data, which may be prone to recall bias and response bias. Additionally, the absence of individual-level data prevented us from conducting stratified analyses by potential effect modifiers such as sex, smoking, and sleep quality. Finally, although we have established a causal link between MDD and IBS, further research is needed to explore the underlying biological mechanisms and potential treatment options.” | Lines 279-287. |
| Discussion  3. The phrase "It is important to prevent IBS among MDD patients at the oneset" contains a typographical error. It should be "onset" instead of "oneset." | Thank you for your kind advice. I have carefully revised the manuscript according to your suggestions. Modification as follows:  “Thus, it is important to prevent IBS among MDD patients at the onset.” | Line 261. |
| Conclusion  1. The discussion effectively emphasizes the clinical implications of understanding the causal relationship between MDD and IBS, advocating for prevention and control strategies. However, explicitly stating the potential clinical impact of the study's findings on patient care and management would provide a more concrete conclusion. | Thank you for your kind advice. I have carefully revised the manuscript according to your suggestions. Modification as follows:  “The research findings have potential clinical significance for the care and management of IBS patients.” | Lines 291-292. |
